# Expert perspectives on the introduction of Triple Artemisinin-based Combination Therapies (TACTs) in Southeast Asia: a Delphi study

**DOI:** 10.1186/s12889-022-13212-x

**Published:** 2022-04-30

**Authors:** Freek de Haan, Wouter P. C. Boon, Chanaki Amaratunga, Arjen M. Dondorp

**Affiliations:** 1 Copernicus Institute of Sustainable Development, Utrecht University, Princetonlaan 8a, 3484 CB Utrecht, the Netherlands; 2 Mahidol Oxford Tropical Medicine Research Unit, Faculty of Tropical Medicine, Mahidol University, Ratchathewi DistrictBangkok, 10400 Thailand

**Keywords:** Malaria, Triple artemisinin-based combination therapies, Drug resistance, Expert perspectives, Delphi study

## Abstract

**Background:**

Triple Artemisinin-based Combination Therapies (TACTs) are being developed as a response to artemisinin and partner drug resistance in Southeast Asia. However, the desirability, timing and practical feasibility of introducing TACTs in Southeast Asia is subject to debate. This study systematically assesses perspectives of malaria experts towards the introduction of TACTs as first-line treatment for uncomplicated falciparum malaria in Southeast Asia.

**Methods:**

A two-round Delphi study was conducted. In the first round, 53 malaria experts answered open-ended questions on what they consider the most important advantages, disadvantages, and implementation barriers for introducing TACTs in Southeast Asia. In the second round, the expert panel rated the relevance of each statement on a 5-point Likert scale.

**Results:**

Malaria experts identified 15 advantages, 15 disadvantages and 13 implementation barriers for introducing TACTs in Southeast Asia in the first round of data collection. In the second round, consensus was reached on 13 advantages (8 perceived as relevant, 5 as not-relevant), 12 disadvantages (10 relevant, 2 not-relevant), and 13 implementation barriers (all relevant). Advantages attributed highest relevance related to the clinical and epidemiological rationale of introducing TACTs. Disadvantages attributed highest relevance related to increased side-effects, unavailability of fixed-dose TACTs, and potential cost increases. Implementation barriers attributed highest relevance related to obtaining timely regulatory approval, timely availability of fixed-dose TACTs, and generating global policy support for introducing TACTs.

**Conclusions:**

The study provides a structured oversight of malaria experts’ perceptions on the major advantages, disadvantages and implementation challenges for introducing TACTs in Southeast Asia, over current practices of rotating ACTs when treatment failure is observed. The findings can benefit strategic decision making in the battle against drug-resistant malaria.

**Supplementary Information:**

The online version contains supplementary material available at 10.1186/s12889-022-13212-x.

## Background

The emergence and rapid spread of antimalarial drug resistance has repeatedly forced malaria endemic countries to adapt their first-line treatment practices for falciparum malaria. These drug transitions have been slow and challenging, even when new therapies were clinically superior to failing alternatives [1–4]. Challenges have been associated with the complex nature of the global health arena and the collective efforts that are required at the global, national, and local-levels [5].

At present, the malaria endemic world relies on artemisinin-based combination therapies (ACTs) for the treatment of uncomplicated falciparum malaria [6]. ACT combines a highly potent, rapidly cleared artemisinin derivative and a less potent, slowly cleared partner drug such as lumefantrine, amodiaquine, piperaquine, pyronaridine or mefloquine. A worrying recent development is multidrug resistance that has emerged to these artemisinin and partner drug combinations and is now spreading through large regions of Southeast Asia [7–9]. In response, policy makers in Cambodia, the country with the highest burden of multidrug-resistant malaria, opt to switch between ACTs when treatment failure is observed [10, 11]. Unfortunately, this strategy of rotating ACTs has proven to be operationally difficult and will likely offer only a temporary remedy before the efficacy of new ACTs also starts to decline [12].

Solutions are required to ensure the continued deployment of effective antimalarial drugs in Southeast Asia and to delay the spread of antimalarial drug resistance to other regions and continents. One promising approach is to complement current ACTs with a third widely used antimalarial drug, creating triple artemisinin-based combination therapies (TACTs) [13]. The rationale is that combining the artemisinin derivative with two partner drugs with counteracting resistance mechanisms will extend the therapeutic lifetime of the drug combinations, because the two partner drugs will provide mutual protection against the development of resistance. Although previous efficacy studies have shown promising results [14], there has been no consensus established yet on the desirability, timing and the practical feasibility of introducing TACTs [13, 15–17]. Little structured data is available on the advantages, disadvantages and implementation challenges for introducing TACTs compared to alternative strategies to address drug-resistant malaria. This study aims to obtain prevailing insights on this important issue. A Delphi study is conducted to map systematically expert perspectives towards the introduction of TACTs compared to applying current strategies of rotating ACTs when treatment failure is observed in Southeast Asia.

## Methods

### Research design

The Delphi technique is a forecasting method that enables exploring implications of multifaceted technological and practical problems [18, 19]. It was developed in the 1950s as a tool for decision-making in situations of insufficient or contradictory information. Delphi studies are iterative in nature and generally comprise two or more rounds of questionnaires with controlled group feedback between each round. In the first round, an expert panel is created and asked to answer open-ended questions regarding an uncertain future. The expert responses are then collected, structured and categorized by the researchers before they are provided back to the same panel. In the second round, the expert panel is asked to rank or rate the inputs of the first round in order to quantify the strength of each statement. More rounds can optionally be included to further validate the findings and to seek expert consensus [20]. The Delphi technique can be modified to meet research goals as long as it includes iterative rounds of data collection with controlled feedback between each round [21, 22]. Delphi studies are generally conducted through online surveys which enables the recruitment of geographically dispersed experts [23].

The Delphi technique facilitates structured communication between experts and allows the inclusion of deviant and minority insights into the collaborative thinking process [18]. Anonymity is an essential feature to avoid conformity and social pressure [24]. The Delphi technique has become a well-established tool in (global) health research [19, 25–28]. Mulligan et al. [29] demonstrated that it is a useful tool for gathering views on research priorities and impact valuations in global health research. The Delphi technique has also proven valuable for assessing decision and economic models in global health [30] and for understanding the dynamics behind the R&D deficit for neglected diseases [31]. This paper uses the Delphi technique to systematically assess perspectives of malaria experts towards the introduction of TACTs in Southeast Asia.

### Expert panelists

Antimalarial drug transitions are complex and multifaceted, involving a wide range of global, national and local-level stakeholders [5]. This multifaceted nature was reflected by purposively selecting experts with different affiliations (e.g. academia, industry, non-governmental organizations, regulators, policy institutes), areas of expertise (e.g. health economics, regulation, market access, malaria drug resistance research), and geographical coverage. An initial list of experts with a track record of relevant expertise was made based on job profiles and published work. This list was then extended by contacting malaria researchers and policy makers in Southeast Asia and asking them to propose additional candidates. The expert list was reviewed by an independent panel and adjustments were made based on their comments. The final list of panelists comprised 146 experts with a balanced representation of affiliations and expertise areas and included experts at multiple geographic locations.

### Software, data security and ethical approval

The *Mesydel* software (https://mesydel.com/en) was used to setup the questionnaires [28, 32], which ensured the essential elements of anonymity, iteration and controlled feedback [22]. The expert panel was approached and invited via automated email to participate in the Delphi exercise. The email included an invitation letter that briefly explained the study objectives and statements on data security and consent. Furthermore, each expert received a unique link to a secured personal survey environment. This was done to grant anonymity and enabled follow-up of non-respondents. Ethical approval for the study was obtained from the Oxford Tropical Research Ethics Committee (OxTREC), reference number: 540–21.

### Delphi procedure, data collection and data analysis

**Fig. 1:**
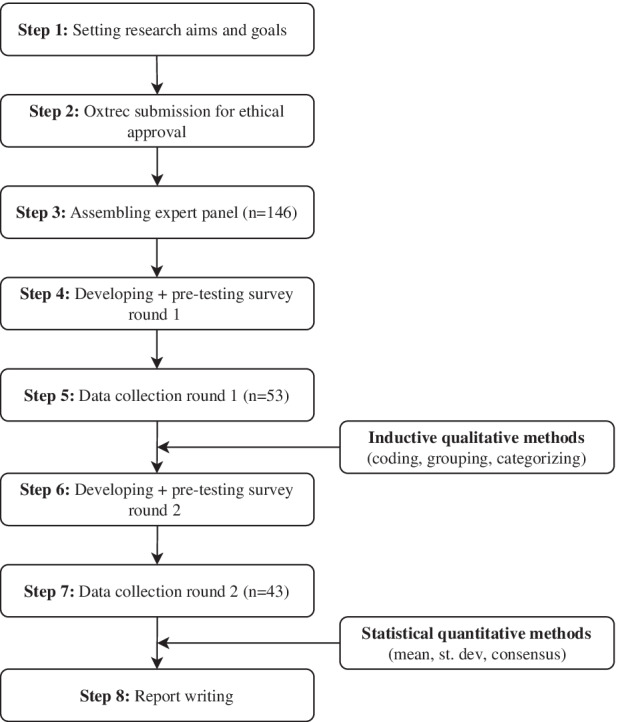
The eight research steps of the two-round Delphi study

A summary of the Delphi procedure is provided in Fig. 1. The first- and second-round questionnaires were developed by the research team and piloted with independent test panels before sending out to the expert panel. The experts were approached via email and reminder emails were sent out at regular intervals to maximize response rates. The questionnaires included sections with demographic questions to gather data on the participants’ background.

#### First round

The first-round questionnaire comprised three sections with open-ended questions. In the first and second section, the expert panel was asked to share what they considered the most important *advantages* and the most important *disadvantages* of introducing TACTs as first-line treatment for uncomplicated falciparum malaria, over current practices of rotating ACTs when treatment failure is observed. In the third section, the expert panel was asked what they considered the most important *implementation barriers* for the introduction of TACTs in Southeast Asia.

The open-ended responses were reviewed using inductive qualitative methods. All statements were de-identified and coded, grouped and categorized by two researchers (FH and CA), first independently and after comparison differences in interpretation were discussed in multiple rounds. After removing duplicates and multiple rounds of analysis and discussion, this resulted in collated lists of 15 advantages, 15 disadvantages and 13 implementation barriers that would serve as input statements for the second round.

#### Second round

The second-round questionnaire was sent to all experts who had responded in the first round. These experts received the collated lists with items and they were asked to rate the *relevance* of each statement on a 5-point Likert scale ranging from ‘highly relevant’ to ‘not relevant’. The following definition for relevance was provided to the experts: ‘Relevance is defined as the expert’s agreement (or disagreement) with the importance of each statement and the extent to which the statement is applicable to TACTs being used in the near future as a replacement to the strategy of rotating ACTs when treatment failure is observed in Southeast Asia’.

Data analysis of the second round involved statistical methods and data visualization techniques performed in Microsoft Excel. We assigned corresponding numbers to each Likert-scale (Highly relevant = 5; Relevant = 4; Somewhat relevant = 3; Slightly relevant = 2; Not relevant = 1) in order to calculate the mean scores and the standard deviation of the expert judgements on each statement. Consensus thresholds were pre-determined at 70%: consensus was reached if 70% of participating experts rated a statement as either ‘highly relevant’,’ relevant’, or ‘somewhat relevant’. Similarly, if 70% of the experts rated the statement as ‘somewhat relevant’, ‘slightly relevant’ or ‘not relevant’ consensus was reached that the statement was not-relevant. The 70% cut-off point has proven to be a useful threshold for determining consensus in several Delphi studies using Likert scales [25, 26, 33].

## Results

First-round data were collected in August and September 2021 and second-round data were collected in October and November 2021. The two rounds directly followed each other in order to keep experts engaged and to maximize response rates. Of the invited 146 experts, 53 completed the first round (36% response rate) and 43 completed the second round (81% response rate). The demographic data of the participating experts is provided in Table 1.

**Table 1:** Demographic data of expert panelists in the first and second round

	Round 1		Round 2	
	N	%	N	%
Gender				
Male	36	68%	31	72%
Female	17	32%	12	28%
Years of relevant work experience				
5–10 years	4	8%	4	9%
> 10—20 years	16	30%	12	28%
> 20 years	33	62%	27	63%
Affiliation^a^				
Academic institution	22	42%	18	42%
Research institution	10	19%	9	21%
Government agency	6	11%	6	14%
Non-governmental organization	12	23%	11	26%
Donor agency	5	9%	3	7%
UN Agency	4	8%	4	9%
Private sector	3	6%	2	5%
Other	4	8%	4	9%
Area of work^a^				
Health economics	3	6%	3	7%
Regulation	3	6%	2	5%
Market access	4	8%	3	7%
Malaria treatment	33	62%	30	70%
Drug development	10	19%	8	19%
Supply chains	4	8%	3	7%
Drug resistance research	24	45%	20	47%
Policy making	12	23%	9	21%
Other	7	13%	4	9%
Affiliated to the DeTACT^b^ project				
No	42	79%	32	74%
Yes	11	21%	11	26%
Country of residence^c^				
Australia	3	6%	2	5%
Bangladesh	1	2%	1	2%
Belgium	1	2%	1	2%
Brazil	1	2%	-	-
Cambodia	4	8%	4	9%
China	2	4%	2	5%
France	1	2%	1	2%
Germany	1	2%	1	2%
Indonesia	3	6%	2	5%
Kenya	1	2%	1	2%
Lao PDR	2	4%	2	5%
Myanmar	5	9%	4	9%
Nigeria	1	2%	1	2%
Philippines	1	2%	-	-
Portugal	1	2%	1	2%
Switzerland	5	9%	5	12%
Thailand	10	19%	8	19%
UK	1	2%	1	2%
USA	8	15%	5	12%
Vietnam	1	2%	1	2%

^a^ Experts could select more than one option for ‘Affiliations’ and ‘Area of work’

^b^ Development of Triple Artemisinin-based Combination Therapies (DeTACT) project

^c^ Some experts do not reside in Southeast Asia yet are involved in malaria treatment practices in the region through international organizations

### First-round results

In the first round of data collection, the participating malaria experts identified a total of 166 advantages, 160 disadvantages and 177 implementation challenges. After grouping, coding and removing duplicates, collated lists of 15 advantages, 15 disadvantages and 13 implementation barriers emerged. The collated lists are provided in Tables 2, 3 and 4, and include brief explanations for each statement and the number of times that each statement was mentioned by individual experts in the first round. These advantages, disadvantages and implementation barriers and the associated brief explanations would serve as input statements for the second-round data collection.

**Table 2:** Expert perspectives on the *advantages* of introducing TACTs over current practices of rotating ACTs when treatment failure is observed in Southeast Asia

Advantages	Explanation	N
Protecting antimalarial drug compounds	TACTs could protect antimalarial drug compounds by preventing parasites from becoming resistant or attaining higher levels of resistance	35
Improving efficacy	TACTs could provide improved antimalarial efficacy and avoid treatment failure	34
Delaying spread of drug resistance	TACTs could prevent or delay the spread of multidrug resistance both locally and to other regions and continents	22
Less frequent policy shifts	TACTs could require less frequent policy shifts and regulatory procedures, which are both time and resource intensive	17
Consistent communication messages	TACTs could allow consistent communication to health workers and patients in terms of work instructions, training and information dissemination	16
Less logistic disruption	TACTs could result in less frequent logistical and operational disruptions in terms of planning, procurement, import, storage and distribution	15
Accelerating malaria elimination	TACTs could accelerate malaria elimination strategies in Southeast Asia	11
Patient/prescriber preference	TACTs’ three-drug compound regimen could be preferred by health workers and patients over the two-drug compound ACT regimen	3
Reducing pressure on surveillance systems	TACTs could mitigate the pressure of monitoring resistance and drug efficacy levels in areas of resistance	3
Reducing malaria transmission	TACTs could contribute to overall reductions in malaria transmission and infections	3
Scaling up production/cost reduction	TACTs could be profitable for pharmaceutical companies by enabling the scale-up of antimalarial drug production and associated cost reductions	2
Regional solution	TACTs could provide a regional solution instead of a solution that needs to be tailored to individual countries	2
Effectivity on vivax malaria	TACTs could contribute in the battle against vivax and other types of malaria and could provide more time to focus on these other types of malaria	1
Prophylactic effect	TACTs could have a malaria prophylactic effect	1
Reduced pill intake	TACTs could reduce the number of pills and/or the days of treatment compared to current ACTs	1

**Table 3:** Expert perspectives on the *disadvantages* of introducing TACTs over current practices of rotating ACTs when treatment failure is observed in Southeast Asia

Disadvantages	Explanation	n
More expensive	TACTs could be more expensive than current ACTs	36
Additional side effects	TACTs could cause additional side-effects such as vomiting, fatigue and headache	25
Unavailability of FDC TACTs	TACTs are not yet available in fixed-dose combinations (FDCs) and FDC product-development timelines could be long	17
Losing drug compounds	TACTs could jeopardize the efficacy of current drug compounds and increase the speed of resistance spreading	14
Toxicity/safety risks	TACTs could increase safety risks, (cardio)toxic effects and negative drug-drug interactions	14
Increasing pill burden	TACTs could have an increased pill burden which may increase the risk of non-compliance	13
Implementation time and costs	TACTs rollout and implementation could be time and resource intensive	11
Limited evidence available	TACTs’ safety and efficacy are not yet scientifically proven	11
Small market size	TACTs could be considered unattractive for pharmaceutical companies because of the limited market size for antimalarials in Southeast Asia	6
Limited timeframe for use	TACTs timeframe for use could be too narrow to warrant the investments in the context of increasing drug resistance and receding falciparum malaria	5
Pharmacovigilance requirements	TACTs implementation could require increased investments in pharmacovigilance systems	3
Reducing sense of urgency	TACTs deployment could reduce the sense of urgency in discovering new drug compounds	2
Limited efficacy	TACTs could have limited clinical response when the individual drug compounds are already failing	1
Limiting credibility of ACTs	TACTs deployment in Southeast Asia could reduce the perceived credibility of ACTs elsewhere	1
Multiple TACTs required	TACTs could not be a 'one size fits all' solution, instead multiple TACTs are required because of a variety in drug resistance profiles	1

**Table 4:** Expert perspectives on the *implementation barriers* for introducing of TACTs in Southeast Asia

Implementation barriers	Explanation	n
Intensified prescriber training	Intensifying training requirements for correct TACTs prescription	27
Donor funder support	Obtaining support by donor funders to cover TACTs implementation costs and potential price increases	24
National policy support	Obtaining support from national malaria control programs and other national decision makers	24
WHO and global policy support	Obtaining support from the WHO and other global decision makers	19
Availability of fixed-dose combination (FDC) TACTs	Ensuring timely development and production of fixed-dose combination (FDC) for TACTs	17
Community acceptance	Ensuring community acceptance by providing clear communication and tackling potential misconceptions about TACTs	12
Collecting safety and efficacy data	Collecting sufficient efficacy and safety data to support the introduction of TACTs	11
Supply chain logistics	Adapting import, procurement and supply routes for the introduction of TACTs	11
Regulatory approval	Obtaining timely regulatory approval for introducing TACTs in Southeast Asia	11
Set up surveillance systems	Setting up surveillance systems to monitor drug resistance and adherence to TACTs	9
Private sector engagement	Engaging the (informal) private sector in TACTs deployment and creating demand beyond official programs	5
Set up pharmacovigilance systems	Setting up a pharmacovigilance system for TACTs	4
Stockpile management	Managing stockpiles for countries that still have ACT stocks or contract deals with ACT producers	3

### Second-round results

**Fig. 2:**
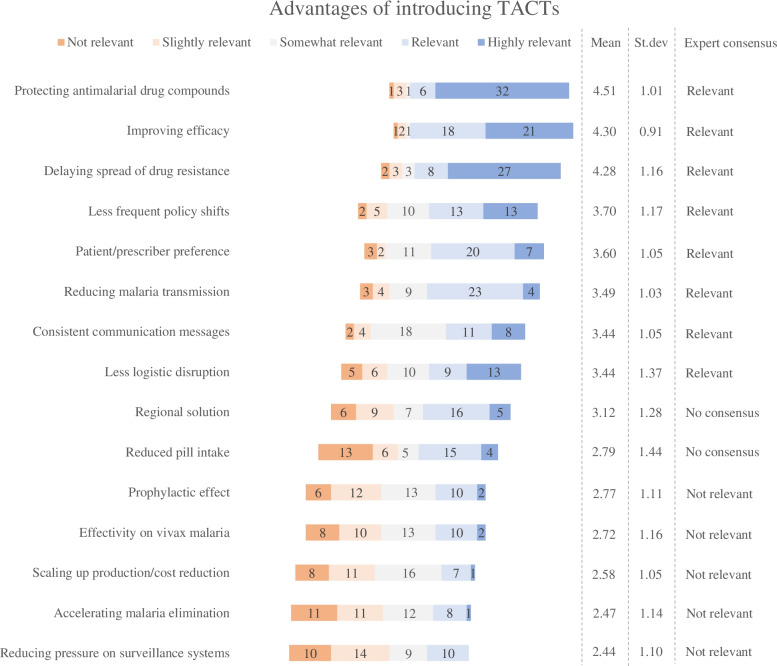
Expert valuations of the advantages for introducing TACTs compared to rotating ACTs. For each item, the mean score, the standard deviation, and the degree of expert consensus are included in the figure. The lists are ranked according to the mean scores of each statement

**Fig. 3:**
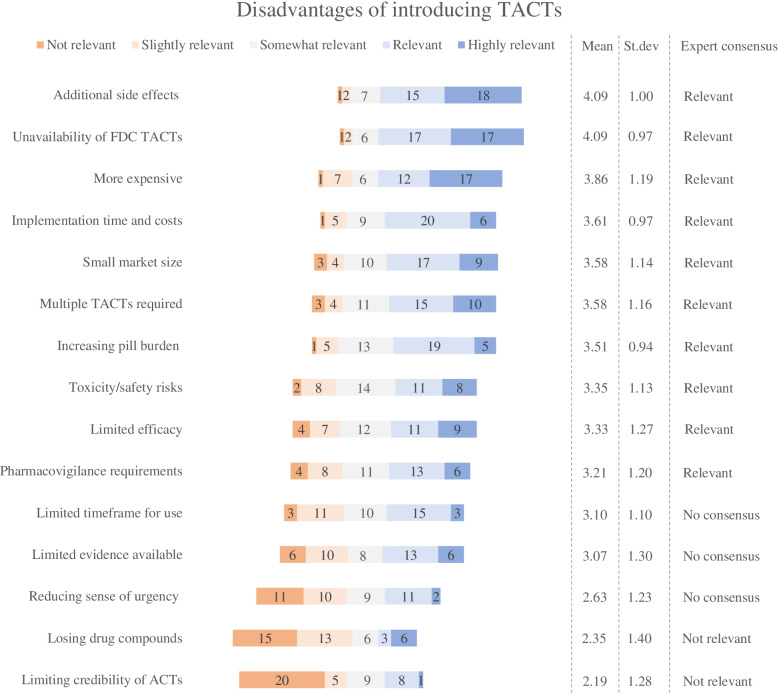
Expert valuations of the disadvantages of introducing TACTs compared to rotating ACTs. For each item, the mean score, the standard deviation, and the degree of expert consensus are included in the figure. The lists are ranked according to the mean scores of each statement

**Fig. 4:**
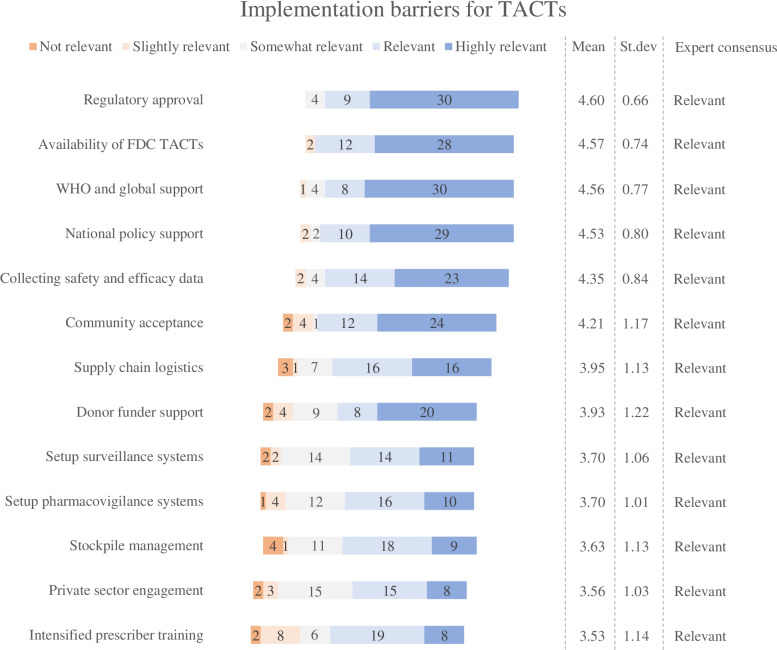
Expert valuations of the implementation barriers for TACTs. For each item, the mean score, the standard deviation, and the degree of expert consensus are included in the figure. The lists are ranked according to the mean scores of each statement

Of the 53 experts that had completed the first round, 43 participated in the second round. Experts reached consensus on 13 advantages, 12 disadvantages and all 13 implementation barriers according to the consensus criteria. On average, the highest scores of experts’ ratings on the 5-point Likert scales were attributed to the implementation barriers (mean score: 4.06) while the average scores of the advantages (mean score: 3.31) and the disadvantages (mean score: 3.30) were nearly identical. Figures 2, 3 and 4 provide the results of the second round of data collection.

#### Advantages of introducing TACTs

The expert panel reached consensus on thirteen advantages for introducing TACTs in Southeast Asia: eight statement were considered to be relevant and five were considered to be not-relevant (Fig. 2). The panel did not reach consensus on two statements. Of the *relevant* statements, the expert panel attributed the highest scores to TACTs’ potential to protect antimalarial drug compounds (mean score: 4.51), its ability to improve efficacy and avoid future treatment failures (mean score: 4.30), and the capacity of TACTs to mitigate the spread of resistance (mean score: 4.28). The same advantages were also mentioned most frequently in the first round, suggesting that the expert panel was consistent in acknowledging TACTs’ potential to overcome the major clinical and epidemiological risks of artemisinin and partner drug resistance.

The expert panel also reached consensus on the relevance of TACTs’ ability to reduce the frequency of policy shifts (mean score: 3.70) and its alignment with patient and prescriber preferences (mean score: 3.60). Of notice, the latter was only mentioned three times as open-text suggestion in the first round and thus represents a minority perspective that gained relevance in the second round. The panel furthermore agreed on TACTs’ potential to reduce malaria transmission and infections (mean score: 3.49), its ability to enable consistent communication messages to prescribers and patients (mean score: 3.44), and the reduced frequency of logistical and operational disruptions that could be instigated by introducing TACTs (mean score: 3.44). The consensus that was achieved on the relevance of these statements indicates that the malaria experts recognize the advantages of introducing TACTs in terms of operational benefits and cost reductions.

Consensus was, however, not reached on the suggested advantages that TACTs could provide a regional solution for the whole of Southeast Asia (mean score: 3.12) and that introducing TACTs may result in a reduced pill intake (mean score: 2.79). The expert disagreement on the relevance of these statements suggest that they consider them as being controversial.

Five statements reached consensus as being *not-relevant*. Unsurprisingly, all five had only been mentioned few times as free-text suggestions in the first round: one expert had cited a prophylactic effect as an expected advantage of introducing TACTs (mean score: 2.77), and one panelist had suggested that TACTs might have advantageous efficacy in vivax malaria (mean score 2.72). The relevance of these statements was rated low, which indicates that the panel either disagrees with their accuracy, or that the panel considered them as only minor advantages. The expert panel rated lowest the advantage of TACTs enabling manufacturers to become profitable by scaling-up production (mean score: 2.58), TACTs’ ability to contribute to accelerated malaria elimination (mean score: 2.47) and the potential of TACTs to mitigate the pressure on surveillance systems in areas of resistance (mean score: 2.44).

#### Disadvantages of introducing TACTs

The expert panel reached consensus on twelve disadvantages for introducing TACTs in Southeast Asia: ten disadvantages were considered to be relevant and two were considered to be not-relevant (Fig. 3). Panelists did not reach consensus on three disadvantages. Of the relevant disadvantages, the expert panel rated additional side-effects for TACTs compared to current ACTs (mean score: 4.09) as highest, emphasizing the importance of such potential adverse effects. High relevance was also attributed to the current unavailability of fixed-dose combinations of TACTs (mean score: 4.09) and concerns of TACTs becoming more expensive than current ACTs (mean score: 3.86). Those items were also among the top three most mentioned disadvantages in the first round, indicating that experts were consistent with their judgement on the relevance of these statements.

Consensus was furthermore reached on disadvantages related to implementation costs and timelines for TACTs (mean score: 3.61), the small market size that could deter drug manufacturers (mean score: 3.58) and concerns that multiple TACTs would be required to address different drug resistance profiles (mean score: 3.58). The latter was only mentioned once as open-text suggestion in the first-round, and thus significantly gained relevance in the second round. The expert panel furthermore agreed on disadvantages related to an increased pill burden for TACTs (mean score: 3.51), and concerns about toxicity and safety risks (mean score: 3.35). Finally, the limited efficacy of TACTs in situations where ACTs are already failing (mean score: 3.33) and the increased pharmacovigilance requirements for TACTs (mean score: 3.21) reached expert consensus as being relevant, despite only being mentioned few times in the first round of data collection.

The expert panel did not reach consensus on four disadvantages that were identified in the first round. They were inconclusive about TACTs’ limited timeframe for use in the context of increasing drug resistance (mean score: 3.10), the limited availability of efficacy and safety evidence (mean score: 3.07), and the reduced sense of urgency that might be instigated by introducing TACTs (mean score: 2.63). The panel reached consensus on two disadvantages as being not relevant. The statement that deploying TACTs could jeopardize the efficacy of current drug compounds (mean score: 3.65) was mentioned by 14 individual experts in the first round but its relevance was rejected in the second round. The expert panel also agreed that introducing TACTs could reduce the perceived credibility of ACTs (mean score: 2.19) was not-relevant; this statement was rated with the lowest mean score of all items.

#### Implementation barriers for TACTs

The expert panel reached consensus on all thirteen implementation barriers, suggesting less ambiguity as compared to the advantages and disadvantages (Fig. 4). There were, however, some notable differences between expert judgements in the first and second round. The panel considered as most relevant implementation barriers: obtaining timely regulatory approval (mean score: 4.60) and ensuring timely availability of fixed-dose combination TACTs (mean score: 4.57). Remarkably, neither of those barriers were among the four most mentioned in the first round of data collection.

Whereas global-level and national-level policy support were proposed equally often as implementation barriers in the first round, subtle differences emerged in their second-round ratings. The expert panel judged the challenges in generating support by the World Health Organization (WHO) and other global decision makers (mean score: 4.56) as slightly more relevant than obtaining support at the national policy levels (mean score: 4.53). Similar high valuations were assigned to the challenges of collecting sufficient safety and efficacy data to support the introduction of TACTs (mean score: 4.35) and the prospective challenges in engaging the community by communicating in a clear way and tackling potential misconceptions about TACTs (mean score: 4.21).

Implementation challenges related to supply chain logistics (mean score: 3.95) and obtaining donor funder support (mean score: 3.93) were rated somewhat lower although the majority of the experts still considered them as relevant barriers. The relatively lower ranking of the latter is noteworthy as it was cited by 24 individual experts in the first round. The setup of surveillance systems to monitor drug resistance and adherence to TACTs (mean score: 3.70) and pharmacovigilance systems (mean score: 3.70) received equal mean scores and were rated slightly higher than challenges related to stockpile management (mean score: 3.63) and engaging private sector actors in a transition to TACTs (mean score: 3.56). Surprisingly, the implementation barrier that was mentioned most often in the first round (27 times) was assigned the lowest relevance in the second round. Still, the relevance of intensified prescriber training (mean score: 3.53) reached expert consensus as being relevant.

## Discussion

### Advantages of introducing TACTs

The expert panel identified 15 advantages that can be grouped into three categories. The first category comprises advantages that are related to the clinical and epidemiological rationale of introducing TACTs in Southeast Asia. Our results indicate that malaria experts do acknowledge that the introduction of TACT is a valid approach to mitigate drug-resistant falciparum malaria, to protect current antimalarial drugs, and to reduce the risk of resistance spreading to other continents and regions. In support of these perspectives are recent studies showing the efficacy of TACTs to treat multidrug-resistant falciparum malaria [14] while further mathematical modelling studies are required to determine its potential in protecting drug compounds and mitigating the spread of resistance [13, 34, 35]. Modeling studies could also inform about implications of introducing TACTs on transmission intensity and on achieving the malaria elimination ambitions in Southeast Asia [36], although the latter was considered to be a not-relevant item by malaria experts in the present study.

The second category of advantages comprises operational advantages and potential cost-reductions as a result of introducing TACTs. Most of the identified benefits in this category can be linked to the scientific rationale of introducing TACTs. For example, the reduced frequency of policy shifts would be a direct consequence of the prolonged therapeutic life time of the antimalarials [16], and the same applies to the benefit of less logistical disruption and consistency of marketing-, and communication messages [11, 37]. In the Delphi exercise, malaria experts acknowledged the relevance of these operational advantages in the context of introducing TACTs. Their perspectives align with literature on previous drug transitions, which have shown that policy shifts [4, 38], logistical disruptions [39] and community awareness [40, 41] require vast investments. Reducing the frequency of drug transitions can therefore mitigate the pressure on scarce financial resources in malaria endemic countries. The expert panel in the present study associated the prospective introduction of TACTs in Southeast Asia with these types of benefits.

The third category of advantages comprises indirect benefits of introducing TACTs. Most advantages in this category were considered to be controversial or their relevance was rejected by the malaria experts. No consensus was, for example, reached on the proposed advantage of reducing the pill burden by introducing TACTs. Neither did the statement that a single TACT can be a regional-wide solution for resistance reach consensus. Indeed, these statements can be considered controversial and to our knowledge, there is no scientific evidence supporting them. The expert panel also assigned low relevance to the post-treatment prophylactic effect of TACTs [34] and to the potential of TACTs to reduce vivax malaria incidence, indicating that experts either disagree with the statements or that they are only considered minor advantages.

### Disadvantages of introducing TACTs

The expert panel identified 15 disadvantages that can be grouped into three categories. The first category comprises statements that relate to acceptance issues. Malaria experts expressed concerns about the potential of adverse effects and other safety risks for TACTs. Indeed, an increase in adverse events such as vomiting, headache and fatigue was also mentioned as a major risk for TACTs’ acceptance in Africa [42]. It is encouraging that clinical studies thus far suggest good tolerability of TACTs, except for a small increased risk of vomiting [14].

Malaria experts also shared concerns that TACTs might become more expensive than current ACTs. Malaria is a poverty-related disease and high consumer prices would likely compromise acceptance [1], especially in private sectors [43, 44]. This emphasizes the importance of donor funder support [45, 46] and alignment with institutional frameworks to improve market prospects [5]. The majority of the expert panel expressed concern that an increased pill burden would negatively affect TACTs’ acceptance. This concern is justified given that in its early days, ACTs were mostly deployed as co-blistered therapies which led to several compliance issues [47, 48], highlighting the importance for TACTs to become available in fixed-dose combinations.

The second category comprises disadvantages that are related to drug development and production deficits. The expert panel voiced concerns about the current unavailability of fixed-dose combinations for TACTs, again emphasizing the importance of combining the triple combinations in one pill [49]. Furthermore, the panelists were concerned that the antimalarial drug market in Southeast Asia may be too small to motivate pharmaceutical companies to pursue TACTs development and production. Similar deficits have been reported repeatedly in the context of pharmaceutical development for malaria [50] and other poverty-related diseases [31, 51]. Encouraging is the growing track-record of successful projects in antimalarial drug development. Public–private partnerships [52–55], regulatory practices [56] and intellectual property management initiatives [57] have contributed to a better incentivized global landscape for pharmaceutical companies to invest in antimalarial drug development and production.

The third category of disadvantages relates to the policy domain. Malaria experts reached consensus that implementation timelines and costs would be a significant disadvantage of TACTs compared to rotating current ACTs [15]. Indeed, introducing a new therapy is time- and resource-intensive [4, 58]. However, these expenses should be considered against the potential costs of more widespread antimalarial drug resistance [59]. No consensus was reached about concerns on the limited timeframe for TACTs deployment in the context of receding malaria in Southeast Asia, revealing this important policy dilemma.

### Implementation barriers for TACTs

The 13 implementation barriers that were identified by experts can be grouped into three categories. The first category relates to challenges in the trajectory toward market introduction of TACTs. The expert panel assigned highest relevance to challenges in obtaining timely regulatory approval for introducing TACTs. This aligns with delays that have been reported in the regulatory trajectory of previous ACTs [54]. The expert panel also attributed high relevance to in-country systems for regulation, including pharmacovigilance-, and surveillance systems [11, 60] and the importance of obtaining sufficient efficacy and safety data to support implementation efforts. Large-scale clinical trials are now underway to obtain such data in order to guide TACTs introduction and deployment [13].

The second category of implementation barriers relates to policy support for TACTs introduction in terms of inclusion in treatment guidelines and implementation programs. The expert panel envisioned challenges in obtaining support at the global policy levels, including WHO and donor funders support. The role of the WHO has been widely acknowledged in other global health transitions [5, 57, 61] and is likely to be essential to the market prospects of TACTs. Experts also considered relevant obtaining national-level policy support to facilitate smooth implementation. Country-level implementation delays were reported in the context of the introduction of ACTs [4] and should be avoided in case TACTs will be introduced. Encouraging are reports from Cambodia, where policy dedication at the national levels and subsequent regulatory and programmatic initiatives have contributed to a successful transition to ACTs in the early 2000s [11, 37, 60].

Community acceptance and logistical challenges, including supply chain management and stockpile management comprise the third category of prospective challenges for introducing of TACTs. Amin et al. (2007) reported that after the shift from monotherapies to ACTs in Kenya, outlets were left with remaining stock of outdated medicines without destination. Other studies have highlighted the importance of adequate and timely supply chain adaptations upon the implementation of new therapies [41]. These studies provide valuable lessons for the potential future implementation of TACTs or other new antimalarial therapies. Experts furthermore agreed that community acceptance could become a challenge towards TACTs deployment, emphasizing the importance of clear communication and marketing messages. Finally, prescriber training [62] and private-sector engagement [2, 60] were cited to be relevant for rapid deployment of TACTs and highlight the need for well-defined implementation strategies.

### Limitations

The Delphi study was designed in adherence to the Conducting and REporting DElphi Studies (CREDES) guidelines [24]. Compared to other Delphi studies, a relative large expert panel was recruited for this Delphi exercise [19, 63] in order to reflect the heterogenous nature of antimalarial drug transitions [5]. Given the spread in the affiliations, areas of expertise and geographical coverage of participating experts (Table 1) and the robustness of second-round findings (Figs. 2, 3 and 4), we consider it unlikely that bias has occurred in the sampling. Still it is possible that non-participating experts would have been able to provide us with complementary insights. At the same time, it is possible that some experts were not able to adequately respond to all the items in the second round. Although experts did have the possibility to leave items unrated, we could have more actively promoted the option to leave items unrated.

Attrition is a known limitation of Delphi studies [20] and attempts were made to minimize attrition rates. We followed-up with non-respondents and conducted both rounds of data collection shortly after each other. This resulted in a relative low attrition of 19% between the two rounds [25, 31]. Anonymity was granted throughout the data collection process to encourage creativity and to promote inclusive views. Some language bias may have occurred since the survey was only conducted in English and for many experts English may not have been their first language.

We are aware that consensus criteria in Delphi studies are subject to interpretation and that using different criteria would likely provide other results in terms of consensus. To avoid bias, we pre-determined cut-off criteria for consensus [19]. Furthermore, we used consensus rates mainly to interpret and to organize results. The goal of this study was to obtain expert perceptions about introducing TACTs. To reflect this objective, we attempted to ask questions in a neutral manner and to provide sufficient explanation with each statement. We explicitly did not aim to create a polling instrument to vote for TACTs nor did we aim to confirm or reject statements. Follow-up research is required to understand the root causes behind the advantages, disadvantages and implementation barriers and to define ways to overcome them.

## Conclusions

The desirability and practical feasibility of introducing TACTs as a response to artemisinin and partner-drug resistance in Southeast Asia is subject of debate. This study systematically assessed perspectives of malaria experts towards the introduction of TACTs as first-line treatment for uncomplicated falciparum malaria in Southeast Asia, over current practices of rotating ACTs when treatment failure is observed. A two-round Delphi study was conducted. In the first round, malaria experts identified 15 advantages, 15 disadvantages and 13 implementation barriers for introducing TACTs in Southeast Asia. In the second round, consensus was reached on 13 advantages (8 perceived as relevant, 5 as not-relevant), 12 disadvantages (10 relevant, 2 not-relevant), and 13 implementation barriers (all relevant). The results of this study add to the limited information available in the public domain to aid in the ongoing debates about strategies to address drug-resistant malaria in Southeast Asia. Policy makers, academic researchers and Non-Governmental Organizations can use the results of this study for prioritizing resources and strategies towards the potential introduction of TACTs.

## Supplementary Information

**Additional file 1.** 

## Data Availability

The de-identified first round data is uploaded as supplementary material. All obtained second round data is included in Table 1 and in Figs. 2, 3 and 4. The full de-identified raw datasets used and/or analyzed during the current study are available from the corresponding author on reasonable request.

## Abbreviations

ACTs: Artemisinin-based Combination Therapies
TACTs: Triple Artemisinin-based Combination Therapies
WHO: World Health Organization
